# A Hybrid UA–CG Force Field for Aggregation Simulation of Amyloidogenic Peptide via Liquid-like Intermediates

**DOI:** 10.3390/molecules30193946

**Published:** 2025-10-01

**Authors:** Hang Zheng, Shu Li, Wei Han

**Affiliations:** 1 School of Chemical Biology and Biotechnology, Peking University Shenzhen Graduate School, Shenzhen 518055, China; 2 Centre for Artificial Intelligence Driven Drug Discovery, Faculty of Applied Sciences, Macao Polytechnic University, Macao 999078, China; shuli@mpu.edu.mo; 3 Department of Chemistry, Hong Kong Baptist University, Kowloon, Hong Kong 999077, China; 4 Institute of Chemical Biology, Shenzhen Bay Laboratory, Shenzhen 518132, China

**Keywords:** hybrid resolution simulation, biomolecular condensates, liquid–liquid phase separation, amyloidogenesis, reaction limited coalescence

## Abstract

Elucidating amyloid formation inside biomolecular condensates requires models that resolve (i) local, chemistry specific contacts controlling β registry and (ii) mesoscale phase behavior and cluster coalescence on microsecond timescales—capabilities beyond single resolution models. We present a hybrid united atom/coarse grained (UA–CG) force field coupling a PACE UA peptide model with the MARTINI CG framework. Cross resolution nonbonded parameters are first optimized against all atom side chain potentials of mean force to balance the relative strength between different types of interactions and then refined through universal parameter scaling by matching radius of gyration distributions for specific systems using. We applied this approach to simulate a recently reported model system comprising the LVFFAR_9_ peptide that can co-assemble into amyloid fibrils via liquid–liquid phase separation. Our ten-microsecond simulations reveal rapid droplet formation populated by micelle like nanostructures with its inner core composed of LVFF clusters. The nanostructures can further fuse but the fusion is reaction-limited due to an electrostatic coalescence barrier. β structures emerge once clusters exceed ~10 peptides, and the LVFFAR_9_ fraction modulates amyloid polymorphism, reversing parallel versus antiparallel registry at lower LVFFAR_9_. These detailed insights generated from long simulations highlight the promise of our hybrid UA–CG strategy in investigating the molecular mechanism of condensate aging.

## 1. Introduction

The spontaneous condensation of biomolecules, such as proteins and nucleic acids, through liquid–liquid phase separation (LLPS) constitutes a fundamental phenomenon in biology [1,2,3]. The condensates formed by these molecules are essentially membrane-less compartments, which play crucial roles in a diverse array of biological processes, including transcription, cellular signaling, and the control of enzymatic reactions [4,5]. While these condensates are essential for normal cellular function, under pathological conditions, certain condensates are metastable and can undergo a pathological conversion into amyloid aggregates composed of disease-associated proteins such as tau, alpha-synuclein, and TDP-43 [6,7,8]. Consequently, understanding the amyloidogenic pathways that operate within these condensates is of paramount biological importance. Although amyloidogenic pathways in dilute solution have long been investigated—and already exhibit considerable mechanistic complexity—the presence of condensates introduces an additional layer of complexity, necessitating the consideration of concurrent changes in both protein structure and the local microenvironment [9]. Elucidating such pathways, particularly during their early stages, remains a significant challenge due to the limited resolution of current experimental characterization methods and the dynamic, heterogeneous nature of fiber formation within condensates [10,11,12,13].

Molecular dynamics (MD) simulations have proven to be a valuable tool for elucidating the molecular details of biomolecular phase separation [14]. However, the application of MD simulations to investigate amyloidogenic pathways within condensates of peptides and proteins has been limited by the absence of suitable simulation models. Computationally expensive, accurate all-atom simulations are often impractical for the timescales required. Coarse-grained (CG) models have been developed to overcome this challenge by simplifying the representation of biomolecules, thereby greatly accelerating sampling [15,16]. Nevertheless, many CG models represent amino acid residues with single beads [17,18,19,20,21,22]. Such residue-level approaches have been widely used to capture homogeneous liquid condensation, but their resolution may limit applicability to more complex amyloidogenic processes occurring within condensates [23]. Other coarse-grained and hybrid-resolution strategies also exist, but a higher level of detail is generally required to describe the underlying protein–protein interactions and conformational transitions [24]. While some CG models have been specifically developed to model amyloid formation, they typically operate by dynamically adjusting interactions between beads based on the presence of neighboring residues. This approach mimics the transformation of experimentally known or hypothesized protein segments from weak binders into stronger beta-sheet binders as a result of conformational changes [25]. Although these models have been successfully used to identify the initial sites of amyloid formation in condensates [26], the actual mechanistic details governing the underlying interactions and conformational transitions remain elusive [27,28].

In this study, we develop a multiscale model to investigate amyloidogenic pathways within condensates. This model is built upon our recently developed framework, which features an atomistic representation for select protein segments and CG representations for the remaining parts of the protein [29,30,31]. It was originally designed to efficiently simulate the conformational dynamics of loop regions using a united-atom (UA) model for peptides while adopting the MARTINI CG representation for structured parts to enhance simulation efficiency. The UA model can capture the details of interactions and conformations and has been calibrated to enable simulations of mini-protein folding and the amyloid formation of key fragments from Aβ and tau [29,30]. Here, we adapt this model for simulating amyloid formation in condensates by representing aggregation-prone regions of proteins with the UA model, while employing MARTINI CG beads to model the remaining parts that presumably participate in less specific interactions. The key to any multiscale model is the identification of proper parameters to describe interactions across different resolutions. We demonstrate that it is possible to obtain such a multiscale model by optimizing these cross-resolution parameters against both the pairwise potential of mean force (PMF) between amino acids and the radius of gyration ($R_{g}$) of peptide monomers—a property extensively employed in the development of CG models for liquid condensates [22,32,33].

We applied our model to simulate a recently reported model peptide, LVFFAR_9_ [34]. This peptide contains an aggregation-prone segment (LVFF) and a polyarginine segment (R_9_) that is responsible for LLPS. Our simulations reveal that these peptides co-assemble with ATP to form droplets. Intriguingly, the LVFF fragments from different chains tend to coalesce into smaller, dynamic clusters that diffuse within the larger droplets. The existence of these specialized architectures was confirmed by our all-atom simulations of a similar, albeit much smaller, system. On a longer timescale, these clusters evolved into amyloid-like structures via internal structural rearrangement. Overall, the multiscale approach proposed here shows great promise for elucidating the mechanistic details of amyloid formation within condensates, offering a valuable computational tool for the study of biomolecular condensates.

## 2. Results and Discussion

### 2.1. Two-Stage Parameter Optimization

**Figure 1:**
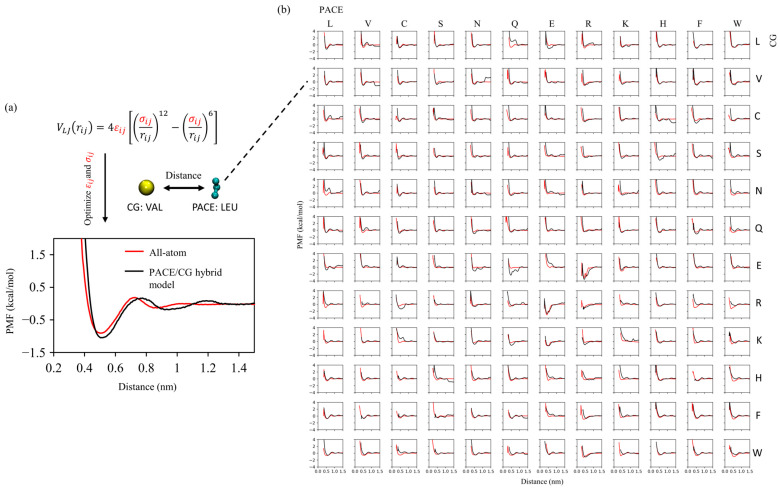
Cross resolution parameterization using side chain analog PMFs. (**a**) Optimization of Lennard–Jones $\varepsilon_{ij}$ and $\sigma_{ij}$ for UA (PACE)–CG (MARTINI) interactions illustrated with the CG Leu–UA Val pair; the optimized hybrid PMF (black) matches the all atom reference (red) in the first well depth and position. (**b**) Matrix of PMFs for all UA–CG residue pairs.

To enable the two resolutions to coexist within the same simulation system, we adopted a two-stage strategy to optimize the interaction parameters. In the first stage, all non-bonded interactions across resolutions were optimized against the pairwise potential of mean force (PMF) between all combinations of sidechain analog dimers. In each dimer system, one side chain was represented with the united-atom model and the other was modeled with MARTINI. For each heterodimer system, two PMFs were considered by swapping their respective resolutions. Figure 1. summarizes all the pairwise PMFs of the dimers from all-atom simulations and compares them with those calculated using our optimized model. Overall, the average deviations in the depth and position of the first free energy minimum are 0.45 kJ/mol and 0.073 nm, respectively. These parameters, optimized at the first stage, lay the foundation for the subsequent system-specific fine-tuning, as discussed next.

In the second stage, the model was fine-tuned for each specific system. For instance, the model peptide LVFFAR_9_ investigated in the present study contains a united-atom level representation for the LVFFA segment and a MARTINI CG level representation [35,36] for the poly-R segment. As shown in previous studies [32,37], the default strength of non-bonded interactions in MARTINI, which was originally calibrated to reproduce the partition coefficients of small molecules between organic and aqueous phases, needs to be re-adjusted to accurately model the LLPS of proteins. The non-bonded interactions must be strong enough to drive LLPS, yet excessively strong interactions can lead to solid-phase separation. To this end, a universal scaling factor, $\epsilon_{CG}$, was optimized and applied to all non-bonded interactions within the CG model. A tractable approach for obtaining such a factor is to optimize it against properties relevant to LLPS. The radius of gyration ($R_{g}$) of a single peptide chain is currently the most widely used metric, as it directly reflects the relative strength of non-bonded interactions between CG beads. More importantly, this property is accessible either experimentally or through all-atom simulations at a reasonable computational cost. This strategy was recently adopted to tune $\epsilon_{CG}$ for modeling the LLPS of FUS and alpha-synuclein using MARTINI [32].

### 2.2. Tuning UA–CG Cross-Interactions via LVFFAR_9_ Monomer $R_{g}$

**Figure 2:**
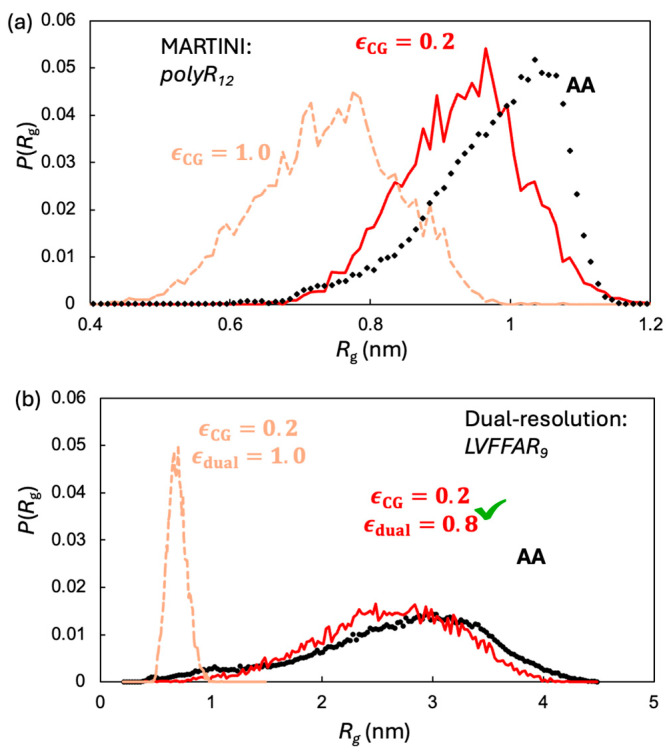
Two stage monomer $R_{g}$ calibration: (**a**) $P(R_{g})$ for R_12_: all atom (black) versus MARTINI with default $\epsilon_{CG}=1.0$ and scaled $\epsilon_{CG}=0.2$, which reproduces the AA peak near ~1.0 nm. (**b**) With $\epsilon_{CG}=0.2$ fixed, the hybrid UA–CG model matches the AA $R_{g}$ distribution of LVFFAR_9_ when cross resolution interactions are scaled to $\epsilon_{dual}=0.8$; without this scaling ($\epsilon_{dual}=1.0$, tan) the monomer is too compact.

While the global scaling factor improved the CG representation, cross-resolution interactions required further refinement. To achieve this, we calibrated the hybrid-resolution LVFFAR_9_ system against the $R_{g}$ of single chains. Specifically, we constructed a system containing a single poly-R (R_12_) chain counterbalanced with ATP molecules. The interactions between ATP and poly-R are responsible for the LLPS of these molecules, as reported previously [34]. The systems were simulated with both the all-atom CHARMM36m force field and the MARTINI model. The $R_{g}$ of R_12_, based on the all-atom simulations, was observed to be concentrated around 1.0 nm, whereas the $R_{g}$ of the same molecule from the CG simulation was significantly smaller, centered at approximately 0.75 nm. This indicates that the default MARTINI non-bonded interactions are too strong to correctly model LLPS. Such an overestimation of interaction strength for LLPS has also been reported for other systems [32,37]. The optimized $\epsilon_{CG}$ for R_12_ was determined to be 0.2, which allows the MARTINI model to accurately reproduce the $R_{g}$ distribution (Figure 2).

Following the approach described above, we introduced another scaling factor, $\epsilon_{dual}$, to fine-tune the parameters of the cross-resolution interactions for the hybrid-resolution LVFFAR_9_ system. Once again, the $R_{g}$ of a single peptide chain obtained from all-atom simulation was employed as the reference. As shown in Figure 2, our optimized $\epsilon_{dual}$ value of 0.8 allows our hybrid-resolution model to correctly reproduce the $R_{g}$ distribution of LVFFAR_9_.

### 2.3. LVFFAR_9_ Co-Assembly into Droplets via Micelle-like Intermediates with LVFF Hydrophobic Core

With the optimized parameters, we employed the hybrid-resolution model to simulate the co-assembly of LVFFAR_9_ with R_9_ and ATP. We first examined a system containing 50 LVFFAR_9_ and 10 R_9_ molecules, corresponding to a composition with approximately 83% LVFFAR_9_, which is beyond the experimentally reported threshold for LVFFAR_9_ to eventually form amyloid aggregates. The combined simulation concentration of LVFFAR_9_ and R_9_ is approximately 27 mM. Although this concentration is significantly higher than the experimental concentration (~1 mM), it serves to accelerate the self-assembly processes, enabling us to observe droplet formation and peptide aggregation within tens of microseconds of simulation time.

**Figure 3:**
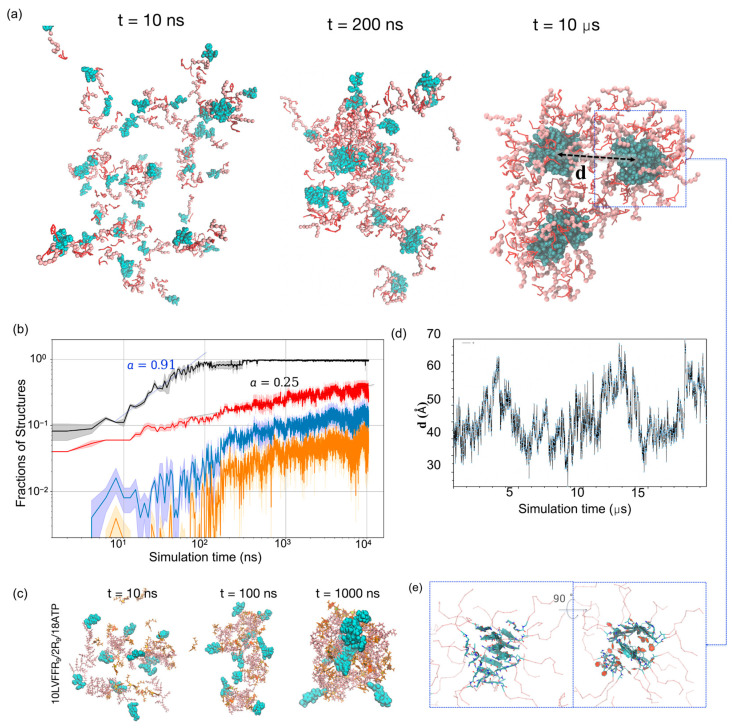
Multistep co assembly and internal amyloidogenesis in LVFFAR_9_/R_9_/ATP mixtures. (**a**) Snapshots at 10 ns, 200 ns, and 10 μs for the 83% LVFFAR_9_ system (50 LVFFAR_9_ + 10 R_9_ + ATP) show rapid droplet formation and micelle-like LVFF clusters (cyan) in a liquid-like matrix; the dashed box highlights the two largest LVFF clusters used in (**d**). (**b**) Kinetics (log–log): fraction of chains in the single largest aggregate (black; early-time n∝tα with α ≈ 0.91, diffusion-limited), fraction of LVFF chains in the largest LVFF cluster (red; α ≈ 0.25, reaction-limited), β-sheet fraction within that cluster (blue), and amyloid-like fraction by registry criteria (orange). Lines are means; shaded bands, s.d.; *n* = 3 runs. (**c**) All-atom reference (CHARMM36m; 10 LVFFAR_9_/2 R_9_/8 ATP) forms micelle-like assemblies on the microsecond scale, supporting the hybrid-model intermediate. (**d**) Center-of-mass distance d between the two largest LVFF clusters in an extended (~20 μs) run shows repeated encounters (~25–35 Å) without fusion, consistent with an activation (electrostatic) coagulation barrier. (**e**) Two orthogonal views of β-tape packing within an LVFF cluster; lateral tape–tape association “sandwiches” Phe rings. (See also Movie S1 for a dynamic visualization of the assembly process).

We then characterized the time evolution of these co-assemblies across three independent 10-μs simulations. LVFFAR_9_ molecules were observed to co-assemble with R_9_ and ATP into spherical aggregates (Figure 3a). We dissected the assembly process by monitoring the time evolution of the structural features of these co-assemblies at different levels of detail. At the molecular level, all three types of molecules rapidly associated with each other to form a single large aggregate at t ≈ 100 ns (Figure 3b). During this process, R9–ATP electrostatic interactions promoted the initial association, while the hydrophobic LVFF fragments segregated to form micelle-like nanostructures, indicating that both types of interactions act synergistically to drive the co-assembly. A dynamic visualization of this process is provided in Movie S1, where LVFF (cyan), R_9_ (gray), and ATP (red) can be clearly distinguished throughout the 20-μs trajectory. A log-log fit of the aggregate size with respect to simulation time indicated that the growth of the aggregate follows a power law, $n∼t^{\alpha }$, with an exponent α close to one, which is characteristic of diffusion-limited coagulation processes [38]. At this stage, the aggregation-prone LVFF fragments, shown as cyan particles in Figure 3a, also began to segregate into small hydrophobic clusters. The formation of such clusters brought together at most 10 LVFFAR_9_ chains, forming micelle-like nanostructures with a hydrophobic core composed of LVFF segments shielded by the R_9_ segments of the peptides. The formation of these micelle-like structures was further confirmed by our all-atom simulations of the co-assembly of a smaller system (Figure 3c). This system, containing only 10 LVFFAR_9_s, 2 R_9_, and a sufficient number of neutralizing ATPs, was found to assemble into a micelle resembling that observed in our hybrid-resolution simulations during a one-microsecond simulation with the CHARMM36m force field. Therefore, the initial stage of the co-assembly process within the first 100 ns resulted in a large aggregate composed of many small micelles, each containing no more than 10 LVFFAR_9_ molecules.

### 2.4. Reaction-Limited Cluster Coalescence and β-Sheet Nucleation

To further probe the maturation of the aggregates, we analyzed the coalescence of LVFF clusters within the droplets. Beyond the initial stage, the size of the largest cluster of LVFF segments continued to grow within the large aggregate (Figure 3b). This indicates that the internal environment of the large aggregate is not solid-like and permits further aggregation of the LVFF clusters. Nevertheless, a log-log fit of the size evolution of the largest LVFF cluster revealed a growth power law with α = 0.25. This suggests that the coagulation of the LVFF clusters is no longer diffusion-limited but is instead severely reaction-limited, requiring the system to overcome an activation barrier for association even when clusters encounter each other via diffusion [39]. To confirm this notion, we extended one of the simulations to 20 μs and monitored the distance between the centers of the two largest LVFF clusters. As shown in Figure 3d, the center-of-mass distance between the two largest LVFF clusters fluctuated over a broad range, from ~25 Å to 70 Å. Importantly, these clusters remained confined within a single large aggregate throughout the trajectory, without de-patching into independent droplets. The repeated approaches to ~25–35 Å followed by separations up to ~70 Å reflect the relative mobility of sub-clusters inside the same condensate. This dynamic behavior suggests that the aggregate interior allows local diffusion and encounters of clusters, consistent with a liquid-like environment, while their failure to merge points to the presence of an electrostatic barrier. During the 20 μs simulation, we observed that the two LVFF clusters came within the encounter distance (~30 Å) on multiple occasions but failed to merge (see Movie S1), supporting the existence of an activation barrier for the coagulation of LVFF clusters. We attribute this coagulation barrier to the electrostatic repulsion between the positively charged outer layers of the micelle-like nanostructures. Similar coagulation barriers have also been reported in previous experimental studies for certain types of colloids [40]. Consistent with this interpretation, our simulations reveal that the R_9_ segments preferentially localize at the micelle periphery, forming a positively charged crown surrounding the hydrophobic LVFF core. This arrangement is expected to generate inter-micelle electrostatic repulsion, thereby hindering further coalescence. Detailed visualizations and quantitative analyses are provided in the Supplementary Information (Movie S1, Figure S1).

Next, we monitored the β-sheet content of the largest growing LVFF clusters (the blue curve in Figure 3b). Notably, we observed a significant increase in β-sheet content once the cluster grew larger than approximately 10 LVFF segments. Thereafter, the β-sheet structures grew steadily as the clusters enlarged, while the fraction of amino acids within the cluster participating in β-sheet structures was maintained at approximately 0.4. This result suggests that the formation of β-sheet structures may not be limited by structural rearrangement within the cluster but is instead governed by the growth of the cluster itself.

### 2.5. Amyloid Registry Polymorphism

We further examined whether the correct packing occurred within the β-sheet structures to give rise to amyloid architectures. Here, amyloid structures were considered to have formed if two neighboring L1VFF4 segments in a β-sheet were aligned in parallel to form L1-L1, V2-V2, F3-F3, and F4-F4 packing, or in an anti-parallel manner to form L1-F4, V2-F3, F3-V2, and F4-L1 packing. Our analysis (see Section 3) revealed that 40% of the β-sheet structures in the largest LVFF clusters were indeed amyloid structures. Interestingly, both parallel and anti-parallel amyloid structures were observed in the co-assembled structures, with the parallel amyloid structure being more favorable (~24% versus ~16%). Inspection of the largest LVFF clusters revealed that the LVFF segments formed multiple β-sheet tapes and that these tapes packed laterally to sandwich the aromatic rings of the phenylalanine residues (Figure 3e). Together, these observations indicate that the hybrid UA–CG model can capture both parallel and anti-parallel registry polymorphism within amyloid β-sheets.

### 2.6. Stoichiometry Dependence of Amyloid Formation

Previous experiments have shown that the LVFFAR_9_-to-R_9_ ratio can influence the co-assembly structures [34]. A lower percentage of LVFFAR_9_ can suppress amyloid formation and increase the stability of the liquid-like aggregates. To further examine whether our hybrid-resolution model could qualitatively capture this composition dependence of co-assembly, we conducted three independent co-assembly simulations with approximately 36% LVFFAR_9_ (i.e., 50 LVFFAR_9_ and 90 R_9_).

**Figure 4:**
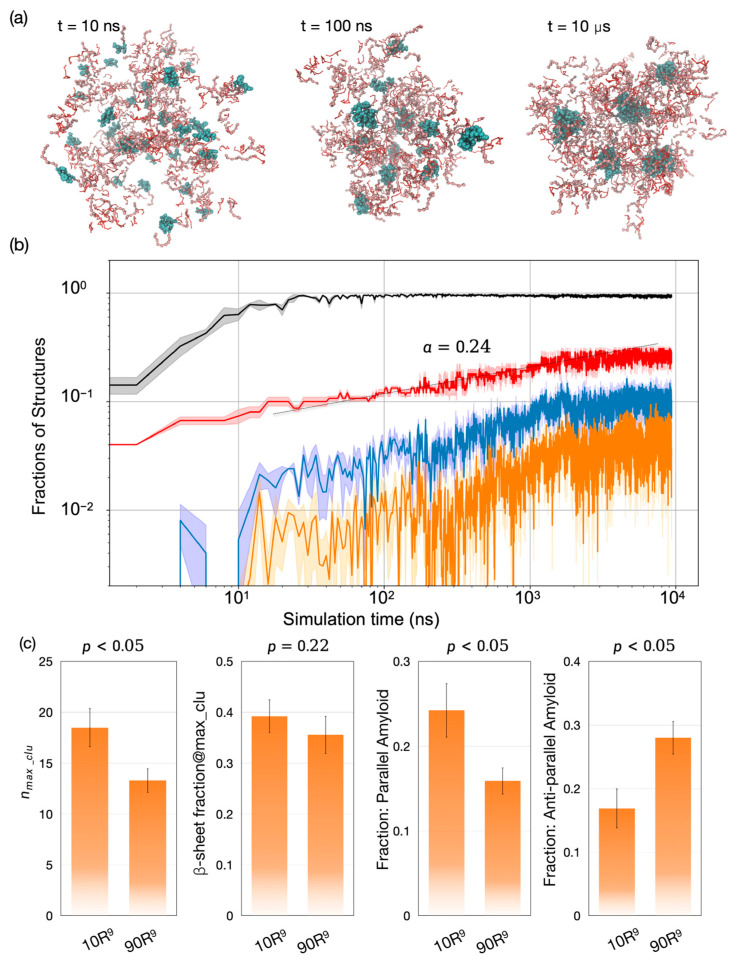
Condensate formation, internal cluster growth, and stoichiometry dependent amyloid polymorphs. (**a**) Representative snapshots for the 83% LVFFAR_9_ mixture (50 LVFFAR_9_+R_9_+ATP) at 10 ns, 100 ns, and 10 μs, showing a single droplet populated by micelle like LVFF clusters (cyan). (**b**) Log–log evolution of structural fractions: chains in the largest overall aggregate (black), LVFF chains in the largest LVFF cluster (red; growth ∝tα with α ≈ 0.24, consistent with reaction limited coagulation), β sheet fraction within that cluster (blue), and amyloid like fraction defined by registry criteria (orange). Curves are means of n = 3 runs; shading denotes s.d. (**c**) End point composition effect: relative to 83% LVFFAR_9_ (“10 R_9_”), the 36% case (“90 R_9_”) shows a smaller largest LVFF cluster size nmax,clu (*p* < 0.05), a similar β sheet fraction (*p* = 0.22), and a reversed amyloid preference-parallel decreases while antiparallel increases (both *p* < 0.05).

Finally, we investigated whether the LVFFAR_9_-to- R_9_ ratio influences the co-assembly pathway. Our simulations revealed a similar process of large aggregate formation via small micelle-like structures (Figure 4a). Again, these large aggregates were formed through diffusion-limited coagulation (black curve, Figure 4b). However, unlike the cluster growth observed at a high percentage of LVFFAR_9_, the cluster growth here was suppressed during the late stage of the simulations (t > 5 μs, red curve in Figure 4b). As a result, the LVFF clusters at the end of these simulations with a lower percentage of LVFFAR_9_ were about 30% smaller than those observed with the higher LVFFAR_9_ percentage. This result is largely in accord with the experimental observation that [34]. The analysis of β-sheet content in the LVFF clusters further revealed that although the LVFF clusters formed at both LVFFAR_9_ percentages exhibited similar amounts of β-sheet content, the preference for amyloid structure types differed between the two conditions. With the lower LVFFAR_9_ percentage, the assembled LVFF cluster preferred the anti-parallel amyloid structures over the parallel ones (28% versus 16%), which is opposite to the trend observed with the high LVFFAR_9_ percentage. This result suggests that the LVFFAR_9_-to-R_9_ ratio affects not only the growth kinetics and the final size of the LVFF clusters within the aggregate but also the subtle structural arrangements within those LVFF clusters.

## 3. Materials and Methods

### 3.1. LVFFAR_9_ Hybrid UA–CG Model

**Figure 5:**
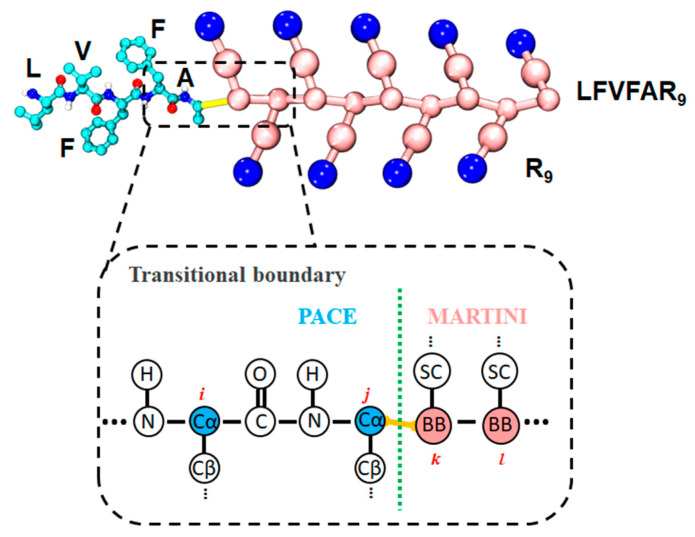
Hybrid UA–CG representation of LFVFAR_9_.

We built the hybrid multiscale model within the same framework established in our previous PACEm framework [41], enabling seamless coupling of UA and CG resolutions under a single simulation protocol. Following this framework, functionally critical region of LVFFA is represented with the PACE (UA) model [30], while the remaining region of R_9_ and the surrounding solvent are modeled with the MARTINI22 CG force field [35], as shown in Figure 5. The total potential is given by $U_{total}=U_{UA}+U_{CG}+U_{UA-CG}$, where $U_{UA}$ follows PACE parameters, $U_{CG}$ follows ElNeDyn22/MARTINI22, and $U_{UA-CG}$ accounts for cross-resolution couplings—comprising bonded terms (bonds and angles) and nonbonded terms (Lennard–Jones (LJ) and Coulombic interactions). The UA terms, the UA–CG bonded couplings, and the Coulombic interactions are identical to our previous PACEm setup [41]. Notably, in contrast to PACEm’s use of an ElNeDyn elastic network [42], the CG region of protein here is modeled with MARTINI22 [35], preserving flexibility at the LVFFAR_9_ C-terminal tail. For UA–CG nonbonded interactions, the Lennard–Jones (LJ) potential was defined as $U_{nonbonded,\;UA-CG,\;LJ}=4\varepsilon_{ij}\left(\frac{\sigma_{ij}^{12}}{r_{ij}^{12}}+\frac{\sigma_{ij}^{6}}{r_{ij}^{6}}\right)$, where $\varepsilon_{ij}$ is the LJ well depth (interaction strength) and $\sigma_{ij}$ is the distance where the LJ potential equals zero. In our previous PACEm parameterization, LJ parameters were assigned via the Lorentz–Berthelot combining rule [43] for simplicity. To better capture specific UA–CG interactions relevant to LVFFAR_9_, we performed pair-specific reparameterization of the cross terms ($\varepsilon_{ij}$, $\sigma_{ij}$) based on PMF matching.

### 3.2. Parameterization of UA-CG LJ Interaction

We computed PMFs for side-chain analog pairs using umbrella sampling to (i) obtain all-atom (AA–AA) reference PMFs and (ii) calibrate UA–CG nonbonded LJ parameters ($\varepsilon_{ij}$, $\sigma_{ij}$). For example, $\varepsilon_{ij}$ and $\sigma_{ij}$ for the interaction between a leucine side-chain UA site and its corresponding CG bead were determined as follows. First, two all-atom leucine side-chain analogs were simulated with a harmonic bias applied to restrain their center-of-mass (COM) separation at a series of windows. The AA–AA PMF was reconstructed with the weighted histogram analysis method (WHAM) as implemented in GROMACS. Next, we simulated a hybrid system in which one leucine side-chain analog was represented at the UA level and the other by its CG bead. Trial LJ parameters ($\varepsilon_{ij}$, $\sigma_{ij}$) were assigned to the UA–CG cross interaction, umbrella sampling was performed using the same windowing protocol, and the resulting hybrid PMF was compared to the AA–AA reference to iteratively optimize $\varepsilon_{ij}$ and $\sigma_{ij}$.

Within the MARTINI 22 force field, the side chains of 18 amino acids (all except glycine and alanine) are represented by CG beads, with several chemistries sharing a common bead type based on physicochemical similarity (e.g., Leu/Ile, Val/Pro, Met/Cys, Asp/Glu, Ser/Thr) [35]. Accordingly, we selected 12 representative side-chain analogs (Leu, Val, Cys, Ser, Asn, Gln, Glu, Arg, Lys, His, Phe, Trp) and fitted UA–CG cross-interaction parameters to reproduce their AA–AA PMFs. Notably, MARTINI2 backbone bead types are defined based on the secondary structure of the backbone. It is noted that MARTINI 22 defines backbone bead types based on the backbone’s secondary structure: N0/Nd/Na for helices, P5 for coils, and Nda for extended structures, excluding Ala and Pro. In our study, the LKVFFR9 peptide adopts a coil structure, with R9’s backbone represented by the P5 bead type. Since the P5 bead type is also used for the MARTINI sidechain particle of Asn, we applied the MARTINI Asn sidechain P5 bead parameters to model interactions between the P5 backbone bead and UA particles.

### 3.3. Simulation Settings

All the simulations were performed with the GROMACS v2018 and v2022 software packages.

We constructed a series of AA systems comprising all pairwise combinations of 12 side-chain analogs (12 × 12 = 144 ordered pairs). Each AA system placed in a 3 × 4 × 3 nm^3^ periodic box filled with TIP3P water [44]. Systems were neutralized with Na+ or Cl− and supplemented to 0.15 M NaCl. Proteins and ions were modeled with the OPLS-AA force field [45]. Short-range nonbonded interactions were truncated at 1.4 nm; long-range electrostatics were treated with Particle Mesh Ewald [46]. After 5000 steps of energy minimization, each system was pre-equilibrated for 0.1 ns in the NPT ensemble with positional restraints on solute heavy atoms. For umbrella-sampling setup [47], one side-chain analog was held positionally restrained while the other was displaced along the y-axis to generate initial configurations at defined COM separations. Umbrella sampling and WHAM [48] were then used to obtain the interaction free energy between the side-chain analogs. The reaction coordinate was the COM distance between the two analogs, restrained with a harmonic bias of 1000 kJ mol^−1^ nm^−2^. For each pair, 20 umbrella windows uniformly spanned a COM distance of 2.5–10 Å, with 20 ns of production sampling per window. Production AA umbrella-sampling runs were conducted with a 2 fs time step at 310 K using the velocity-rescale thermostat and at 1 bar using isotropic Parrinello–Rahman coupling.

For mixed-resolution systems, the protein was modeled using the hybrid UA–CG force field, while water and NaCl were represented using the MARTINI2 model. A 1.2 nm cutoff was applied to both LJ and Coulombic interactions; electrostatics beyond the cutoff were treated with a reaction-field potential. Each system underwent 5000 steps of energy minimization followed by 1 ns of NPT pre-equilibration, during which positional restraints on solute atoms were gradually released. Production runs of LVFFAR_9_ were conducted without restraints at 310 K using the v-rescale thermostat and at 1 bar using the Parrinello–Rahman barostat with isotropic coupling and a compressibility of 4.5 × 10^−5^ bar^−1^. Hydrogen mass repartitioning enabled a 4 fs time step [49]. Umbrella sampling settings (reaction coordinate, force constant, window spacing, per-window sampling) matched those used in the AA simulations, and PMFs were reconstructed with WHAM.

All files required to reproduce the system setup and production simulations with the hybrid-resolution (UA–CG) model—including topologies, coordinates, UA–CG coupling parameter tables, and GROMACS/analysis control files—are openly available at: https://github.com/hanlab-computChem/hanlab/tree/master/UA_CG_LLPS_Amyloid (accessed on 28 September 2025).

## 4. Conclusions

In this study, we developed a hybrid-resolution model to investigate the mechanistic details of amyloid formation within biomolecular condensates. Our model integrates a united-atom representation for aggregation-prone segments with the MARTINI coarse-grained model for the remaining protein regions. We demonstrated a two-stage parameterization strategy, optimizing cross-resolution interactions first against all-atom potentials of mean force and then refining the model against the radius of gyration of the peptide, which establishes a robust protocol for developing such multiscale models.

The application of this model to the co-assembly of the LVFFAR_9_ peptide with R_9_ and ATP revealed a multi-step aggregation pathway. We observed an initial, rapid formation of a large, spherical aggregate through a diffusion-limited coagulation of smaller, micelle-like nanostructures. These intermediates, which consist of a hydrophobic LVFF core shielded by a polyarginine corona, were found to be a key structural feature of the early assembly process for peptides like LVFFAR_9_ that contain both aggregation-prone and LLPS-prone segments. The subsequent growth of the LVFF clusters within the larger aggregate was found to be a significantly slower, reaction-limited process. Our analysis suggests this slowdown is due to an activation barrier for the fusion of the micelle-like structures, which we attribute to electrostatic repulsion between their charged surfaces.

Furthermore, our simulations at a hybrid resolution captured the emergence of ordered β-sheet structures, which occurred only after the LVFF clusters grew beyond a critical size of approximately 10 peptides. We further examined the influence of stoichiometry on the assembly process. Simulations performed at a lower LVFFAR_9_-to-R_9_ ratio showed that while the overall aggregation mechanism was similar, the growth of LVFF clusters was kinetically suppressed. This compositional change also led to a notable shift in the preferred amyloid packing, favoring anti-parallel β-sheet arrangements over the parallel structures observed at higher LVFFAR_9_-to-R_9_ ratios.

The results presented here show that our hybrid-resolution approach can successfully capture the complex interplay between phase separation and amyloid aggregation, from the formation of initial condensates to the development of specific amyloid polymorphs. The model provides molecular-level insights into the intermediates and kinetic barriers that govern amyloidogenesis within a condensed phase. Although this model may rely on the parameter refinement against monomeric radius of gyration for each specific peptide system, this procedure is straightforward and manageable. Overall, our hybrid-resolution approach offers a valuable computational framework for studying the mechanisms of pathological protein aggregation.

## Data Availability

All files required to reproduce the system setup and production simulations with the hybrid-resolution (UA–CG) model—including topologies, coordinates, UA–CG coupling parameter tables, and GROMACS/analysis control files—are openly available at: https://github.com/hanlab-computChem/hanlab/tree/master/UA_CG_LLPS_Amyloid (accessed on 28 September 2025).

## Supplementary Materials

The following supporting information can be downloaded at: https://www.feishu.cn/mail/page/attachment?token=XTwPbC0o5oboFqxicZscASUGnic accessed on 28 September 2025.

## Abbreviations

The following abbreviations are used in this manuscript:

AA	Institute All-atom
CG	Coarse-grained
UA	United-atom
LLPS	Liquid–liquid phase separation
$R_{g}$	Radius of gyration
PMF	Potential of mean force
WHAM	Weighted histogram analysis method
ATP	Adenosine triphosphate
